# Jasmonate ZIM-Domain (JAZ) Protein Regulates Host and Nonhost Pathogen-Induced Cell Death in Tomato and *Nicotiana benthamiana*

**DOI:** 10.1371/journal.pone.0075728

**Published:** 2013-09-27

**Authors:** Yasuhiro Ishiga, Takako Ishiga, Srinivasa Rao Uppalapati, Kirankumar S. Mysore

**Affiliations:** Plant Biology Division, The Samuel Roberts Noble Foundation, Ardmore, Oklahoma, United States of America; University of Louisville, United States of America

## Abstract

The nonhost-specific phytotoxin coronatine (COR) produced by several pathovars of *Pseudomonas syringae* functions as a jasmonic acid-isoleucine (JA-Ile) mimic and contributes to disease development by suppressing plant defense responses and inducing reactive oxygen species in chloroplast. It has been shown that the F-box protein CORONATINE INSENSITIVE 1 (COI1) is the receptor for COR and JA-Ile. JASMONATE ZIM DOMAIN (JAZ) proteins act as negative regulators for JA signaling in *Arabidopsis*. However, the physiological significance of JAZ proteins in *P. syringae* disease development and nonhost pathogen-induced hypersensitive response (HR) cell death is not completely understood. In this study, we identified *JAZ* genes from tomato, a host plant for *P. syringae* pv. *tomato* DC3000 (*Pst* DC3000), and examined their expression profiles in response to COR and pathogens. Most *JAZ* genes were induced by COR treatment or inoculation with COR-producing *Pst* DC3000, but not by the COR-defective mutant DB29. Tomato SlJAZ2, SlJAZ6 and SlJAZ7 interacted with SlCOI1 in a COR-dependent manner. Using virus-induced gene silencing (VIGS), we demonstrated that SlJAZ2, SlJAZ6 and SlJAZ7 have no effect on COR-induced chlorosis in tomato and *Nicotiana benthamiana*. However, *SlJAZ2*-, *SlJAZ6*- and *SlJAZ7*-silenced tomato plants showed enhanced disease-associated cell death to *Pst* DC3000. Furthermore, we found delayed HR cell death in response to the nonhost pathogen *Pst* T1 or a pathogen-associated molecular pattern (PAMP), INF1, in *SlJAZ2*- and *SlJAZ6*-silenced *N. benthamiana*. These results suggest that tomato JAZ proteins regulate the progression of cell death during host and nonhost interactions.

## Introduction

Several pathovars of *Pseudomonas syringae*, including pvs. atropurpurea, glycinea, maculicola, morsprunorum and tomato, produce the nonhost-specific phytotoxin coronatine (COR). COR is a polyketide formed by the coupling of coronafacic acid (CFA) and coronamic acid (CMA) through an amide bond [1]. COR functions as a structural and functional analog of a phytohormone, jasmonic acid-isoleucine (JA-Ile) [2], [3], [4]. Furthermore, the F-box protein CORONATINE INSENSITIVE 1 (COI1) is known to be required for COR and JA signaling in tomato and *Arabidopsis* [1], [2], [5], [6]. COR activates the JA-signaling pathway by mimicking JA-Ile in *Arabidopsis* and tomato and thereby functions to suppress the stomata-mediated and/or SA-mediated defenses, thus allowing bacteria to grow to higher densities *in planta* [6], [7], [8], [9]. However, the physiological significance of COR-induced disease development is not completely understood.

Recently, we demonstrated that loss-of-function of *SGT1* (a suppressor of the G2 allele of *skp1*) abolished COR-induced chlorosis and showed a reduction of disease-associated cell death and chlorosis induced by inoculation with *P. syringae* pv. *tomato* DC3000 (*Pst* DC3000), suggesting a connection between COR-induced chlorosis and cell death [10]. In addition, we revealed that COR targets the photosynthetic machinery to modulate chloroplastic reactive oxygen species (ROS) homeostasis in promoting disease-associated cell death during bacterial speck disease of tomato [11], [12], [13], [14]. We demonstrated that the loss-of-function of *NADPH-dependent thioredoxin reductase C* (*NTRC*), an electron donor for peroxiredoxins (Prxs) in the NADPH-dependent thioredoxin (Trx) system, leads to accelerated ROS accumulation resulting in enhanced disease symptoms in tomato and *Arabidopsis* upon inoculation with *Pst* DC3000, indicating that ROS detoxifying systems, including NTRC/Prxs, function as negative regulators of disease-associated cell death [14]. However, physiological and molecular understanding of localized cell death during *Pst* DC3000-induced disease development is still unknown. It is important to understand these processes for engineering bacterial speck resistance in tomato and to better understand other plant-pathogen interactions.

JA-Ile/COR receptor COI1 functions as a central component in the JA-signaling pathway in *Arabidopsis* and tomato [2], 5,6,9,15. In the presence of JA-Ile or COR, COI1 interacts with JASMONATE ZIM DOMAIN (JAZ) proteins, which function as repressors and form a receptor complex. After forming a receptor complex, JAZ proteins are ubiquitinated by the SCF (Skip1/Cullin1-F-box) ubiquitin ligase SCF^COI1^ complex and degraded through the 26 proteosome [16]. In addition, JAZ proteins interact with key transcription factors (TFs) for JA-inducible genes, such as MYC2-class TFs, and repress the expression of these genes by making a repressor complex with NOVEL INTERACTOR OF JAZ (NINJA) and TOPLESS (TPL) proteins [17]. Thus, the expression of JA-inducible genes is activated by the MYC2-class of TFs in response to JA-Ile or COR.

In this study, we examined the physiological significance of JAZ proteins in *Pst* DC3000 disease development and nonhost pathogen-induced cell death in tomato and *Nicotiana benthamiana*. Most tomato *JAZ* genes (*SlJAZ*) showed significant induction in response to COR treatment and inoculation with a COR-producing pathogen. Silencing of *SlJAZ2*, *SlJAZ6* and *SlJAZ7* genes enhanced disease-associated cell death induced by *Pst* DC3000. Furthermore, we demonstrated that *SlJAZ2*- and *SlJAZ6*-silenced *N. benthamiana* had delayed HR in response to nonhost pathogens. Therefore, our results indicate that tomato JAZ proteins modulate the progression of cell death during both host and nonhost pathogen interactions.

## Materials and Methods

### Plant Materials, Bacterial Strains and Inoculation Methods

Tomato (*Solanum lycopersicum* cv. Glamour) and *Nicotiana benthamiana* plants were maintained in the greenhouse under a 14 h light/10 h dark photoperiod at 25±2°C. The tomato seedlings assay was used for expression analysis of the *SlJAZ* genes [18]. Briefly, tomato seeds were germinated and maintained on 1/2 Murashige and Skoog (MS) medium (0.3% phytagel) with Gamborg vitamins (PhytoTechnologies Laboratories, Shawnee Mission, KS, USA) and used for assay 4 days after germination at 25°C in darkness. Tomato seedlings were treated with water as a mock control or 200 pmol of COR (Sigma-Aldrich, St. Louis, MO, USA). Tomato seedlings were inoculated by a method whereby MS agar plates were flooded with bacterial suspension as described previously [18]. To perform uniform inoculation, the bacterial suspension (OD600 = 0.1) containing 0.025% Silwet L-77 (OSi Specialties Inc., Danbury, CT, USA) was dispensed into Petri dishes until the seedlings were completely submerged, and the seedlings were exposed to bacterial cells for 2–3 min with gentle agitation. After the inoculum was discarded, Petri dishes containing inoculated seedlings were incubated with a light intensity of 200 µE m-2 sec-1 and a 12 h light/12 h dark photoperiod.

*Pseudomonas syringae* pv. *tomato* DC3000 (*Pst* DC3000) was used as the wild-type strain for virulence assays on tomato. The COR-defective mutant *Pst* DB29 was previously described [19] and was kindly provided by Dr. Barbara Kunkel (Washington University, St Louis, USA). *P. syringae* pv. *tomato* T1 (*Pst* T1) was used as the nonhost pathogen on *N. benthamiana*. The strains of *Pst* DC3000, *Pst* DB29 and *Pst* T1 were grown at 28°C on mannitol-glutamate (MG) medium [20] containing antibiotics in the following concentrations (µg ml−1): rifampicin, 25; kanamycin, 50; and spectinomycin, 25 for 36–48 h. Prior to inoculation, bacteria were suspended in 1 ml sterile distilled H_2_O.

Pathogen inoculation assays on vector control and VIGS-silenced tomato were conducted as described [8]. Briefly, bacterial suspensions (OD600 = 0.1) were prepared in sterile distilled water containing 0.025% Silwet L-77 (OSI Specialties Inc., Danbury, CT, USA) and spray-inoculated to runoff. The inoculated plants were then incubated in growth chambers at ∼100% RH for the first 24 h and at ∼70% RH for the rest of the experimental period. The inoculated plants were observed for 7 dpi for symptom development. Bacterial growth in leaves was measured by determining the internal bacterial populations. Prior to sampling, leaves were surface-sterilized with 15% H_2_O_2_ for 3 min to eliminate epiphytic bacteria and then washed with sterile distilled water. The leaves were then homogenized in sterile distilled water, and serial dilutions were plated onto MG medium containing antibiotics. The bacterial population at 0 dpi was estimated from leaves harvested 1 h post inoculation. Bacterial growth was evaluated in three independent experiments.

To analyze the impact of nonhost pathogen-induced HR cell death in wild-type and *SlJAZs*-silenced *N. benthamiana* plants, bacterial suspensions (OD600 = 0.05) of *Pst* T1 were prepared and infiltrated into leaves using a 1 ml needleless syringe. HR cell death was observed 24 hours after infiltration.

### Virus-induced Gene Silencing and *Agrobacterium*-mediated Transient Expression Assay

*Agrobacterium tumefaciens* GV2260 containing *pTRV1* and *pTRV2* with a gene of interest (e.g., *pTRV2*::*GFP*, *pTRV2*::*SlJAZ2*, *pTRV2*::*SlJAZ6*, and *pTRV2*::*SlJAZ7*) were grown overnight on LB medium containing antibiotics (rifampicin, 25 µg/ml; kanamycin, 50 µg/ml) at 28°C. Bacterial cells were harvested and resuspended in induction medium (10 mM MES pH 5.5; 200 µM acetosyringone) and incubated at room temperature on an orbital shaker for 5 h. The bacterial cultures containing *pTRV1* and *pTRV2* with the gene of interest were mixed in equal ratios (OD600 = 0.5) and infiltrated into lower leaves using a 1 ml needleless syringe [21]. The infiltrated plants were maintained in the greenhouse and used for studies 15–21 days post-infiltration.

To analyze the impact of PAMP-mediated HR in wild-type and *SlJAZs*-silenced plants, *A. tumefaciens* GV2260 containing 35S::*INF1* within the T-DNA of a binary vector was grown overnight on LB medium containing antibiotics (rifampicin, 25 µg/ml; kanamycin, 50 µg/ml) at 28°C. Bacterial cells were harvested and resuspended in induction medium and incubated at room temperature in an orbital shaker for 12 h. *Agrobacterium* cultures (OD600 = 0.8) were infiltrated into leaves using a 1 ml needleless syringe. Four days after infiltration, HR cell death was observed.

### Real-time Quantitative RT-PCR

Total RNA extraction and real-time quantitative RT-PCR (qRT-PCR) were done as described previously [11]. Total RNA was extracted using TRIzol (Sigma-Aldrich) according to manufacturer’s protocol. Total RNA was treated with Turbo DNase (Ambion, Austin, TX, USA) to eliminate genomic DNA, and 5 µg of DNase-treated RNA was reverse transcribed using Superscript III reverse transcriptase (Invitrogen, Carlsbad, CA, USA) with oligo d(T)15–20 primers (Invitrogen). The cDNA (1∶20) was then used for qRT-PCR that was performed using primers shown in Table S1 with Power SYBR Green PCR master mix (Applied Biosystems, Foster City, CA, USA) on an ABI Prism 7900 HT sequence detection system (Applied Biosystems). The *N. benthamiana* and tomato *Actin* genes were used as internal controls. To study the downregulation of transcripts in silenced plants, qRT-PCR primers that anneal outside the region targeted for silencing were used (Table S1). Average CT values calculated using Sequence Detection Systems (version 2.2.2; Applied Biosystems) from triplicate samples were used to determine the fold expression relative to controls.

### Yeast Two-hybrid Analysis

The Matchmaker yeast two-hybrid system from Clontech (Takara Bio, Kyoto, Japan) was used according to manufacturer’s protocols. Briefly, full-length cDNA for tomato genes, including *SlCOI1*, *SlJAZ1*, *SlJAZ2*, *SlJAZ3*, *SlJAZ4*, *SlJAZ5*, *SlJAZ6*, *SlJAZ7*, *SlJAZ8*, *SlJAZ9*, *SlJAZ10*, *SlJAZ11*, *SlJAZ12* and *SlNINJA*, were amplified by RT-PCR with specific primer sets (Table S1) and then were inserted into the yeast expression vectors *pGBKT7* (bait) and *pGADT7* (prey) using In-Fusion® HD Cloning Kit from Clontech (Takara Bio). To determine the interactions between fusion proteins, both bait and prey plasmids were co-transformed into MaV203 yeast strain (Invitrogen) carrying three GAL4-inducible reporter genes (*lacZ*, *HIS3* and *URA3*). Bait-prey interactions were selected on the synthetic dropout medium lacking Leu and Trp (SC-Leu-Trp, Sigma-Aldrich). The yeast colony grown in SC-Leu-Trp was streaked on the medium lacking Leu, Trp and His (SC-His-Leu-Trp, Sigma-Aldrich) supplemented with 10 mM 3-amino-1,2,4-triazole (Sigma-Aldrich) with or without COR (20 µg/mL).

### Ion Leakage Measurements

Ion leakage is used as an indirect quantitative measure for the disease-associated cell death. Ion leakage was measured as described previously [11]. Six leaf discs were punched from *N. benthamiana* leaves inoculated with *Pst* T1 and *A. tumefaciens* carrying 35S::*INF1*, and agitated in 30 ml of distilled water for 3 h; electrical conductivity was measured using an ion conductivity meter (Orion 555A, Thermo Electron Corp., Marietta, OH, USA). Plants were then autoclaved for 20 min to kill the cells and release total ions into the medium. Values relative to the whole ion content after autoclaving were used to express the percent ion leakage.

## Results and Discussion

### Tomato *SlJAZ* Genes were Upregulated by Coronatine and *Pseudomonas syringae* pv. *tomato* DC3000

**Figure 1:**
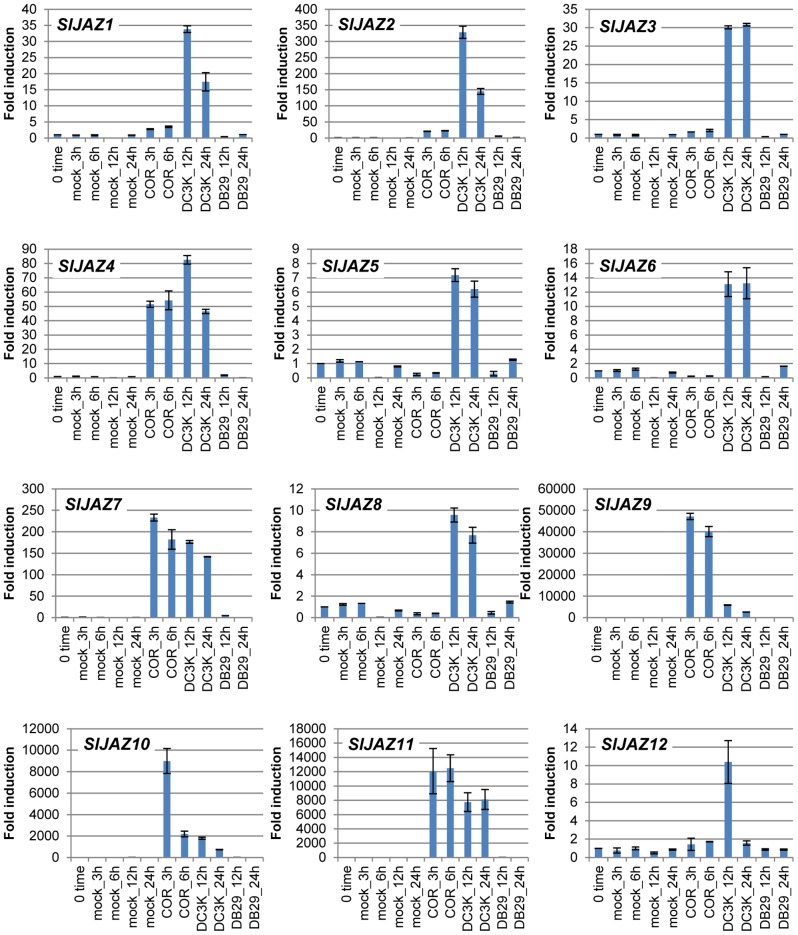
Expression profiles of *JAZ* genes in tomato seedlings inoculated with *P. syringae* pv. *tomato* DC3000 (*Pst* DC3K) or the COR-defective mutant *Pst* DB29, or treated with COR. Tomato seedlings were treated with distilled water (mock control), COR (20 pmol) or inoculated with *Pst* DC3K or *Pst* DB29. The expression of genes encoding tomato JAZs was evaluated by real-time quantitative RT-PCR. Bars represent the means ± standard deviation (SD).

It has been reported that there are 12 members of *JAZ* family genes in *Arabidopsis* and tomato [22], [23](Fig. S1 and Table S2). In *Arabidopsis*, it was demonstrated that the expression of *AtJAZs* was upregulated during *Pst* DC3000 infection in a COR-dependent manner [24]. To examine the expression profiles of tomato *SlJAZs* during *Pst* DC3000 infection, tomato seedlings were treated with water or COR, or inoculated with *Pst* DC3000 or the COR-defective mutant *Pst* DB29, and qRT-PCR analysis was performed. Based on previous reports for *JAZ* family genes in tomato [23], we developed gene specific primer sets for 12 tomato *SlJAZ* genes including *SlJAZ1* (Solyc07g042170), *SlJAZ2* (Solyc12g009220), *SlJAZ3* (Solyc03g122190), *SlJAZ4* (Solyc12g049400), *SlJAZ5* (Solyc03g118540), *SlJAZ6* (Solyc01g005440), *SlJAZ7* (Solyc11g011030), *SlJAZ8* (Solyc06g068930), *SlJAZ9* (Solyc08g036640), *SlJAZ10* (Solyc08g036620), *SlJAZ11* (Solyc08g036660) and *SlJAZ12* (Solyc01g009740) (Table S1 and S2). The expression of all *SlJAZ* genes was induced by *Pst* DC3000 infection (Fig. 1). The expression of *SlJAZ3*, *SlJAZ5*, *SlJAZ6*, *SlJAZ8* and *SlJAZ12* was not induced after treatment with purified COR (Fig. 1). In contrast, *SlJAZ1* and *SlJAZ2* were moderately induced, and *SlJAZ4*, *SlJAZ7*, *SlJAZ9*, *SlJAZ10* and *SlJAZ11* were highly induced by purified COR (Fig. 1). It has been reported that the expression of *JAZ* was activated by JA-induced degradation of JAZ proteins in *Arabidopsis* and rice [25], [26]. We demonstrated that 12 *SlJAZ* genes were induced by *Pst* DC3000, but only seven of 12 *JAZ* genes were upregulated in response to COR. In *Arabidopsis*, most of the *JAZ* genes were induced within 30 min after JA treatment [26]. In this study, we examined the expression of *SlJAZs* at 3 hours after treatment with purified COR, suggesting that the expression of *SlJAZ3*, *SlJAZ5*, *SlJAZ6*, *SlJAZ8* and *SlJAZ12* could be transiently induced within 3 hours after treatment with purified COR.

Together with other studies in *Arabidopsis*, our results suggest that the expression of *JAZ* genes was differently regulated by environmental stimuli such as pathogen infection, wounding and herbivory [24], [27]. It has been reported that only eight of 12 *JAZ* genes were upregulated during *Pst* DC3000 infection in *Arabidopsis* [24]. We demonstrated that all *JAZ* genes are significantly induced by *Pst* DC3000 in tomato (Fig. 1). Although the JA-mediated signaling pathway is critical for the pathogenicity of *Pst* DC3000 in *Arabidopsis* and tomato, different sets of genes were expressed during *Pst* DC3000 infection between *Arabidopsis* and tomato [18], [28]. Therefore, it is tempting to speculate that *Pst* DC3000 uses different strategies to modulate disease development by regulating different *JAZ* genes during pathogenesis between tomato and *Arabidopsis*.

To investigate whether COR is the virulence factor responsible for the expression of *JAZ* genes in tomato, we examined the COR-defective mutant *Pst* DB29 and found that the induction of *JAZ* genes in tomato is COR-dependent (Fig. 1). Consistent with our results, it has been reported that COR is the primary virulence factor inducing the expression of *JAZ* genes in *Arabidopsis* [24]. These results clearly indicate that the COR-induced JA-signaling pathway has a critical role in the pathogenicity of *Pst* DC3000 in both *Arabidopsis* and tomato.

### SlJAZ2, SlJAZ6 and SlJAZ7 Interact with SlCOI1 in a Coronatine-dependent Manner

**Figure 2:**
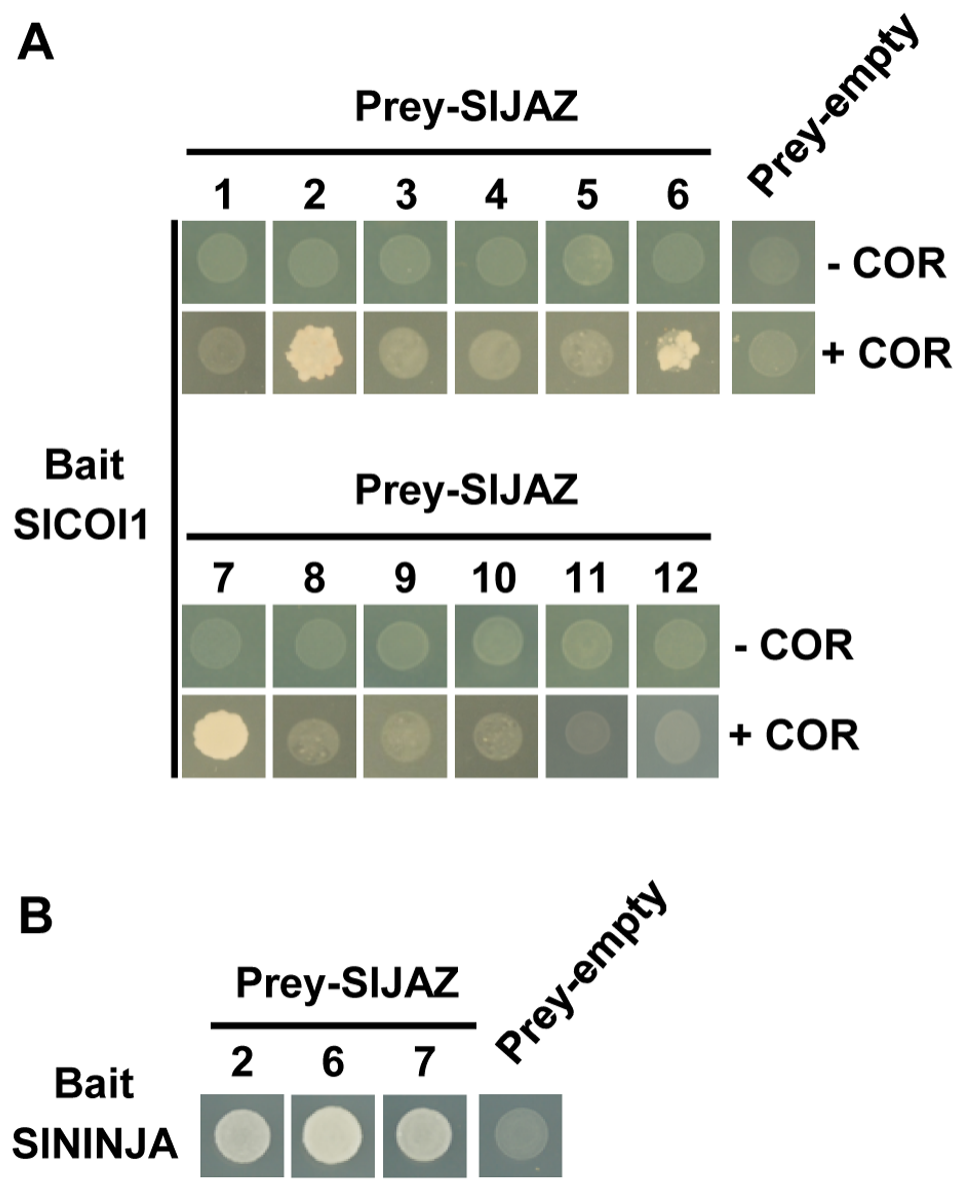
COR-dependent interaction between SlCOI1 and SlJAZs. (**A**) Interaction between SlCOI1 and SlJAZs. (**B**) Interaction between SlNINJA and SlJAZs. The MaV203 yeast strain carrying the construct for SlCOI1 (bait) and SlJAZs (prey) was grown on synthetic dropout (SD) glucose medium without Leu, Trp and His in the absence or presence of COR (20 µM). Photographs were taken at 5 days.

In previous studies, it was demonstrated that some JAZ proteins interact with COI1 to form a receptor complex for JA-Ile/COR in *Arabidopsis*, tomato and rice [5], [25], 29,30. To investigate whether SlJAZ proteins form a receptor complex with SlCOI1, we cloned full length ORFs for 12 *SlJAZs* (Fig. S2) and examined the interaction between SlJAZs and SlCOI1 using the yeast two-hybrid system. Consistent with a previous report [5], SlJAZ2 and SlJAZ6 interacted with SlCOI1 in a COR-dependent manner (Fig. 2A). In addition to SlJAZ2 and SlJAZ6, we found SlJAZ7 interacted with SlCOI1 in the presence of COR, suggesting that SlJAZ2, SlJAZ6 and SlJAZ7 could form a receptor complex for JA signaling in tomato (Fig. 2A). Unlike *Arabidopsis*, most SlJAZ proteins failed to interact with SlCOI1 in yeast two-hybrid system. This could be due to low expression of these SlJAZ proteins in yeast. The use of other methods for protein interactions, such as pull-down assay, may provide clues to the interactions between SlCOI1 and SlJAZ proteins.

It has been demonstrated that JAZ proteins also interact with the adaptor protein NINJA and form a repressor complex together with TOPLESS in *Arabidopsis* [17]. To investigate whether SlJAZ proteins form a repressor complex with SlNINJA or not, we cloned full-length ORF for *SlNINJA* (Fig. S2) and examined the interaction between SlJAZs and SlNINJA. SlJAZ2, SlJAZ6 and SlJAZ7 interacted with SlNINJA (Fig. 2B), suggesting that SlJAZ2, SlJAZ6 and SlJAZ7 could form a repressor complex to regulate JA-inducible genes.

### Silencing of *SlJAZ2*, *SlJAZ6* and *SlJAZ7* in Tomato and *Nicotiana benthamiana* Plants had no Effect on COR-induced Chlorosis

Since SlCOI1 interacted with tomato JAZ proteins SlJAZ2, SlJAZ6 and SlJAZ7 (Fig. 2A), we determined the requirement of these JAZ proteins for COR-induced chlorosis using virus-induced gene silencing (VIGS). One of the hallmarks of bacterial speck disease on tomato leaves is the formation of necrotic lesions surrounded by chlorosis. COR is known to induce chlorosis in a COI1-dependent manner [14]. Since JAZ proteins are known to function as repressors, we investigated whether silencing of *SlJAZs* leads to hypersensitivity to COR. We treated vector control (TRV::*GFP*) and *SlJAZs*-silenced tomato and *N. benthamiana* plants with 200 pmol of COR and observed COR-induced chlorosis. To avoid off-target effect of VIGS, we selected gene specific region as the trigger sequence, which does not have 21 nucleotide homology to other *SlJAZs*. The efficiency of silencing was confirmed by the quantification of the endogenous transcript levels using qRT-PCR (Fig. S3). As shown in Fig. S4, there was no significant difference on COR-induced chlorosis between control and *SlJAZs*-silenced plants in tomato and *N. benthamiana*, indicating that SlJAZ2, SlJAZ6 and SlJAZ7 do not play an active role in COR-induced chlorosis. It is possible that these JAZ proteins have functional redundancy in COR-induced chlorosis. It was reported that *Atjaz10* mutant showed hypersensitivity to JA, since alternative splice variants functioned as the stable repressors of transcription [31]. Although SlJAZ2, SlJAZ6 and SlJAZ7 have no high homology to AtJAZ10, further investigation about alternative splice variants should help us to better understand the function of SlJAZs in COR-induced chlorosis.

### Silencing of *SlJAZ2*, *SlJAZ6* and *SlJAZ7* Enhances Disease Development caused by *Pseudomonas syringae* pv. *Tomato* DC3000

**Figure 3:**
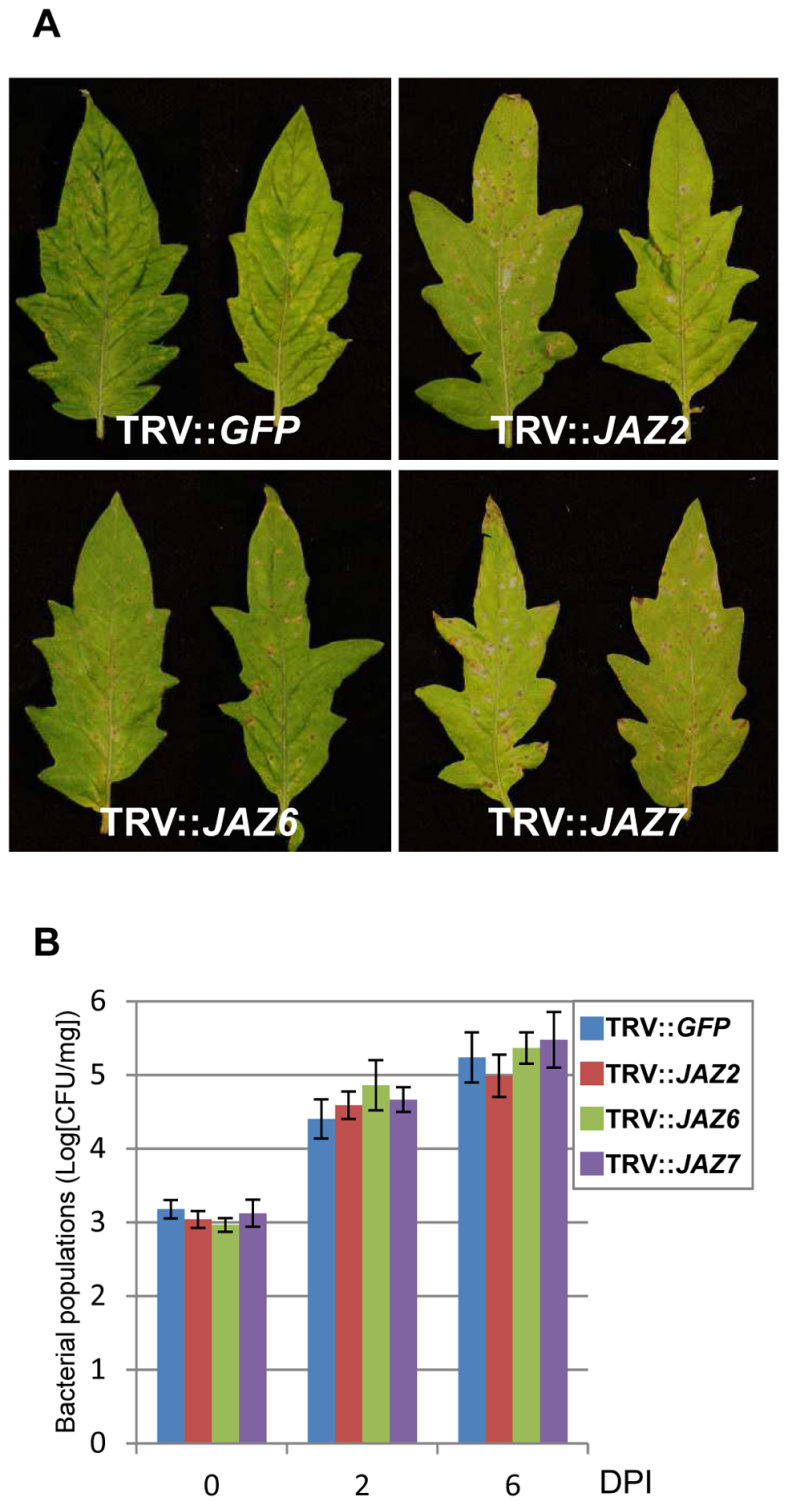
Disease development by *Pseudomonas syringae* pv. *tomato* DC3000 in *SlJAZs*-silenced tomato plants. (**A**) Bacterial speck of tomato on control and *SlJAZs*-silenced tomato leaves induced by *P. syringae* pv. *tomato* DC3000. The control (TRV::*GFP*) and *SlJAZs*-silenced (TRV::*SlJAZ2*, TRV::*SlJAZ6* and TRV::*SlJAZ7*) tomato leaves were spray-inoculated with *P. syringae* pv. *tomato* DC3000 (5×10^7^ CFU/ml), and photographs were taken at 7 days after inoculation. (**B**) Bacterial populations of spray-inoculated control (TRV::*GFP*) and *SlJAZs*-silenced (TRV::*SlJAZ2*, TRV::*SlJAZ6* and TRV::*SlJAZ7*) tomato leaves at 2 and 6 dpi. Error bars represent standard deviations of six replicates.

To determine whether *SlJAZ2*, *SlJAZ6* and *SlJAZ7* play a role in disease symptom development induced by *Pst* DC3000, we spray-inoculated control (TRV::*GFP*) and *SlJAZs*-silenced tomato plants with *Pst* DC3000. The control plants developed typical bacterial speck symptoms with necrotic lesions surrounded by chlorotic halos (Fig. 3A). However, *SlJAZ2*-, *SlJAZ6-* and *SlJAZ7*-silenced plants developed accelerated necrotic lesions with reduced chlorotic halos (Fig. 3A). Interestingly, no significant differences in bacterial multiplication were observed between the control and *SlJAZs*-silenced plants at 2 and 6 days after inoculation (Fig. 3B). In previous study, we have demonstrated that silencing of *NTRC* and *Prx* resulted in accelerated necrotic lesions during *Pst* DC3000 infection without increasing the bacterial growth [14]. In *Arabidopsis*, it has been reported that the JA-mediated signaling pathway leading to disease development is distinct from the signaling pathway regulating the bacterial multiplication [32]. Therefore, these results indicated that the disease phenotypes in *SlJAZs*-silenced plants might be due to hypersensitivity to COR and JAZ proteins may function as a negative regulator of disease-associated cell death without altering the pathogen multiplication.

It has been reported that the *Arabidopsis Atjaz10* mutant has enhanced susceptibility to *Pst* DC3000 [24]. Consistent with our results, they demonstrated that AtJAZ10 affects disease development without increasing the bacterial populations [24]. Together, these results indicate that some members of JAZ proteins may function as negative regulators of disease development during *Pst* DC3000 infection in tomato and *Arabidopsis*.

In a previous report, we demonstrated that silencing of *THYLAKOID FORMATION1* (*THF1*) in *N. benthamiana* and tomato produced a necrosis-like phenotype to COR application. *THF1* silenced tomato and *Arabidopsis thf1* mutant showed enhanced necrotic lesions with reduced chlorosis in response to *Pst* DC3000 [33]. As shown in Fig. 3A, *SlJAZ2*-, *SlJAZ6* and *SlJAZ7*-silenced plants also developed enhanced necrotic lesions. Therefore, we speculate that JAZ protein may function as a negative regulator of disease development by modulating *THF1*.

### Silencing of *SlJAZ6* and *SlJAZ7* Delays Nonhost Pathogen- and PAMP-mediated HR Cell Death

**Figure 4:**
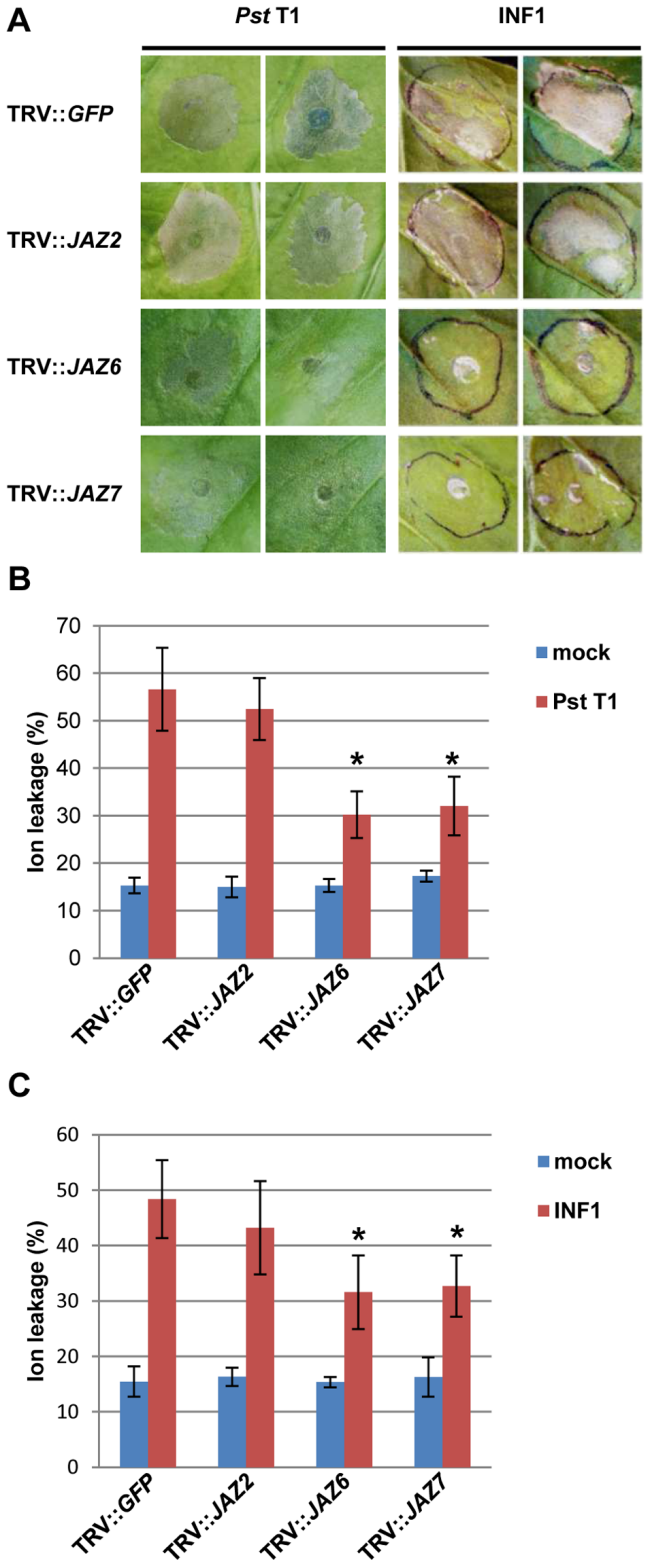
Nonhost pathogen- and PAMP-mediated hypersensitive response cell death in *SlJAZs*-silenced *N. benthamiana* plants. (**A**) HR cell death induced by nonhost pathogen *P. syringae* pv. *tomato* T1 and *A. tumefaciens* carrying INF1 on control and *SlJAZs*-silenced *N. benthamiana* leaves. The control (TRV::*GFP*) and *SlJAZs*-silenced (TRV::*SlJAZ2*, TRV::*SlJAZ6* and TRV::*SlJAZ7*) *N. benthamiana* leaves were syringe-inoculated with nonhost pathogen *P. syringae* pv. *tomato* T1 (5×10^7^ CFU/ml) and *A. tumefaciens* that can express *INF1 in planta*. The photographs were taken at 24 hours (*Pst* T1) and 3 days (INF1) after inoculation. Ion leakage from control and *SlJAZs*-silenced *N. benthamiana* leaves after inoculation with nonhost pathogen *P. syringae* pv. *tomato* T1 (**B**) and *A. tumefaciens* that can express *INF1 in planta* (**C**). Values show the percentage of total ions. Vertical bars indicate the standard error for three independent experiments. *Asterisks* indicate a significant difference from the control (*GFP*) using a *t-test* (* = *P*<0.05).

To further investigate the functional role of JAZ in plant-microbe interactions, we examined nonhost pathogen- and PAMP-mediated hypersensitive response (HR) cell death in *JAZs*-silenced *N. benthamiana* plants. To determine whether JAZs affect HR cell death, we syringe-infiltrated control (TRV::*GFP*) and *JAZs*-silenced *N. benthamiana* plants with the nonhost bacterial pathogen *P. syringae* pv. *tomato* T1 and *A. tumefaciens* that can express 35S-*INF1 in planta*. The control and *JAZ2*-silenced plants showed HR cell death in response to nonhost pathogen and INF1 at 24 hpi and 3 dpi, respectively (Fig. 4). Interestingly, *JAZ6-* and *JAZ7*-silenced plants delayed HR cell death compared with control plants, suggesting that JAZ6 and JAZ7 may have a role in nonhost pathogen- and PAMP-mediated signaling pathways leading to HR cell death (Fig. 4).

It has been demonstrated that the *N. benthamiana* JAZ protein NbPPS3, which has a high similarity to SlJAZ5, has a role in the PAMP-mediated signaling pathway leading to HR cell death [34]. Katou et al. reported that NbPPS3 protein was phosphorylated by both SIPK and WIPK in response to PAMP. Silencing of *NbPPS3* delayed PAMP-mediated HR cell death, indicating that NbPPS3 is one of the downstream components of the MAPK-mediated signaling pathway leading to HR cell death [34]. In addition, Oh et al. reported that silencing of *Nicotiana attenuata JAZ* gene *NaJAZh*, which has a high similarity to *SlJAZ3*, showed spontaneous cell death associated with the accumulation of salicylic acid and hydrogen peroxide in the leaves [35]. Therefore, it is likely that the COI1-JAZ complex and/or other components of JA/COR signaling pathways play a role during HR cell death.

## Conclusion

In this study, we identified a role for JAZs in host and nonhost pathogen-induced cell death. Although the precise role of JAZs in the COR/JA signaling pathway and cell death needs further investigation, our results present a new role for JAZs in bacterial speck disease development. We also demonstrated that JAZ proteins have important roles in cell death during nonhost pathogen infection. It has been demonstrated that JAZ proteins interact with the MYC2-class of TFs to modulate JA-inducible genes in *Arabidopsis*. However, the TFs targeted by JAZ are still unclear in tomato. Further identification of other TFs targeted by tomato JAZs and characterization of their mode of action in conjunction with COR are needed to understand the mechanisms of JAZs during host and nonhost pathogen interactions.

## Supporting Information

Figure S1 **Phylogenetic tree of the** ***JAZ*** **family genes in** ***Arabidopsis*** **and tomato.**
(TIF)

Figure S2 **Sequences of full-length ORFs for 12 tomato** ***SlJAZ*** **and** ***SlNINJA.***
(PDF)

Figure S3 **Determination of silencing efficiency of** ***JAZ2, JAZ6*** **and** ***JAZ7*** **in tomato and** ***N. benthamiana*** **.** **A.** Relative transcript levels of *JAZs* in tomato. B. Relative transcript levels of *JAZs* in *N. benthamiana*. The *N. benthamiana* and tomato *Actin* were used as internal controls.
(TIF)

Figure S4 **COR-induced chlorosis on control and** ***SlJAZs*** **-silenced tomato and** ***N. benthamiana*** **plants.** Purified COR was applied to vector control (TRV::*GFP*) or *SlJAZs*-silenced (TRV::*SlJAZ2*, TRV::*SlJAZ6* and TRV::*SlJAZ7*) tomato and *N. benthamiana* leaf tissues in 2 µl aliquots (2 nM), and a visible chlorotic zone was scored at 4 days after treatment.
(TIF)

Table S1 List of primers used in this study.
(XLSX)

Table S2 List of *JAZ* family genes in tomato.
(XLSX)
