# Supplementary figures and images for: Jasmonate ZIM-Domain (JAZ) Protein Regulates Host and Nonhost Pathogen-Induced Cell Death in Tomato and Nicotiana benthamiana
Source: PLoS One. 2013 Sep 27;8(9):e75728. doi: 10.1371/journal.pone.0075728 (PMC3785428; doi:10.1371/journal.pone.0075728)

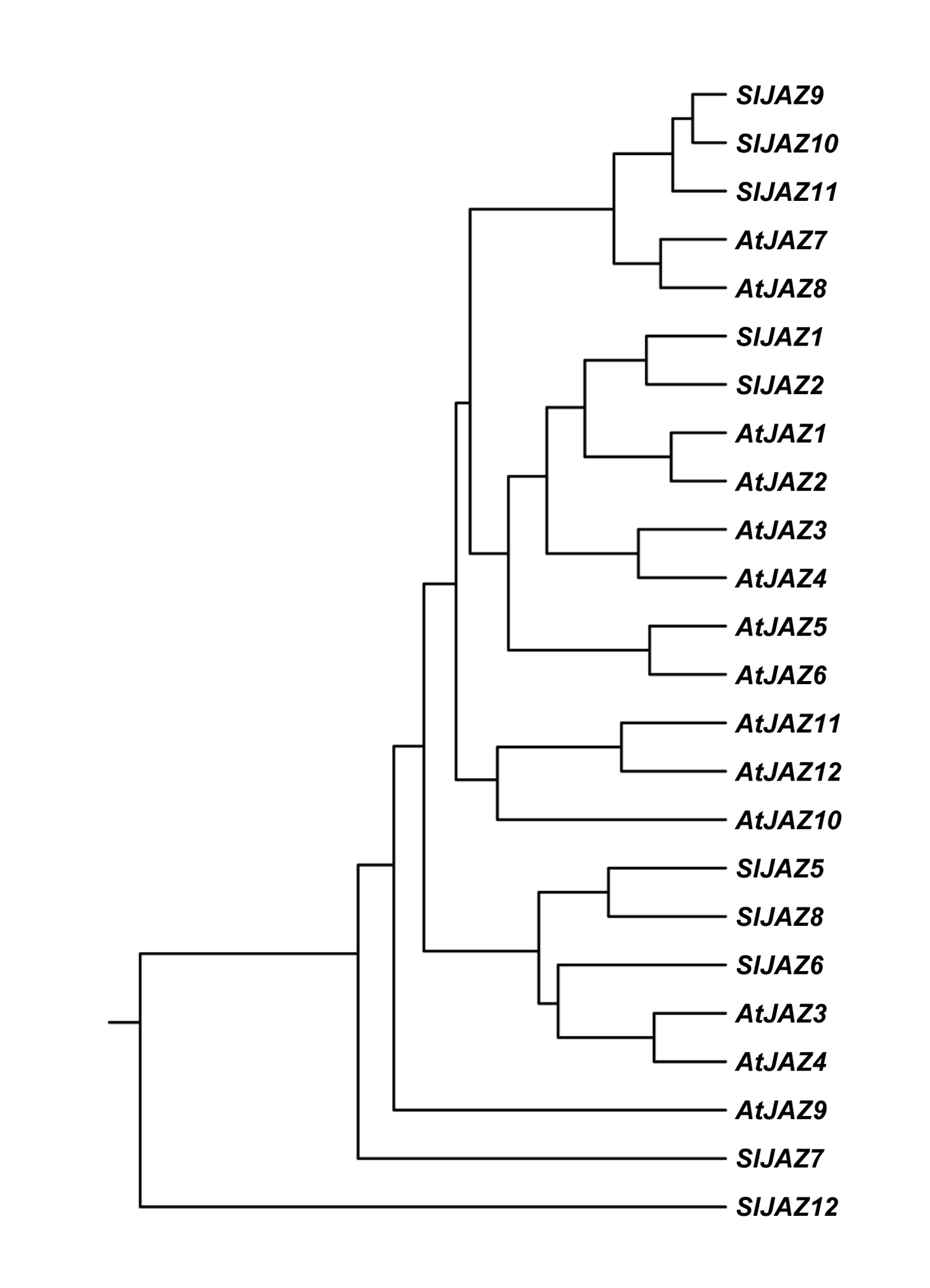

Supplement: Figure S1 — Phylogenetic tree of the JAZ family genes in Arabidopsis and tomato. (TIF) [file pone.0075728.s001.tif]

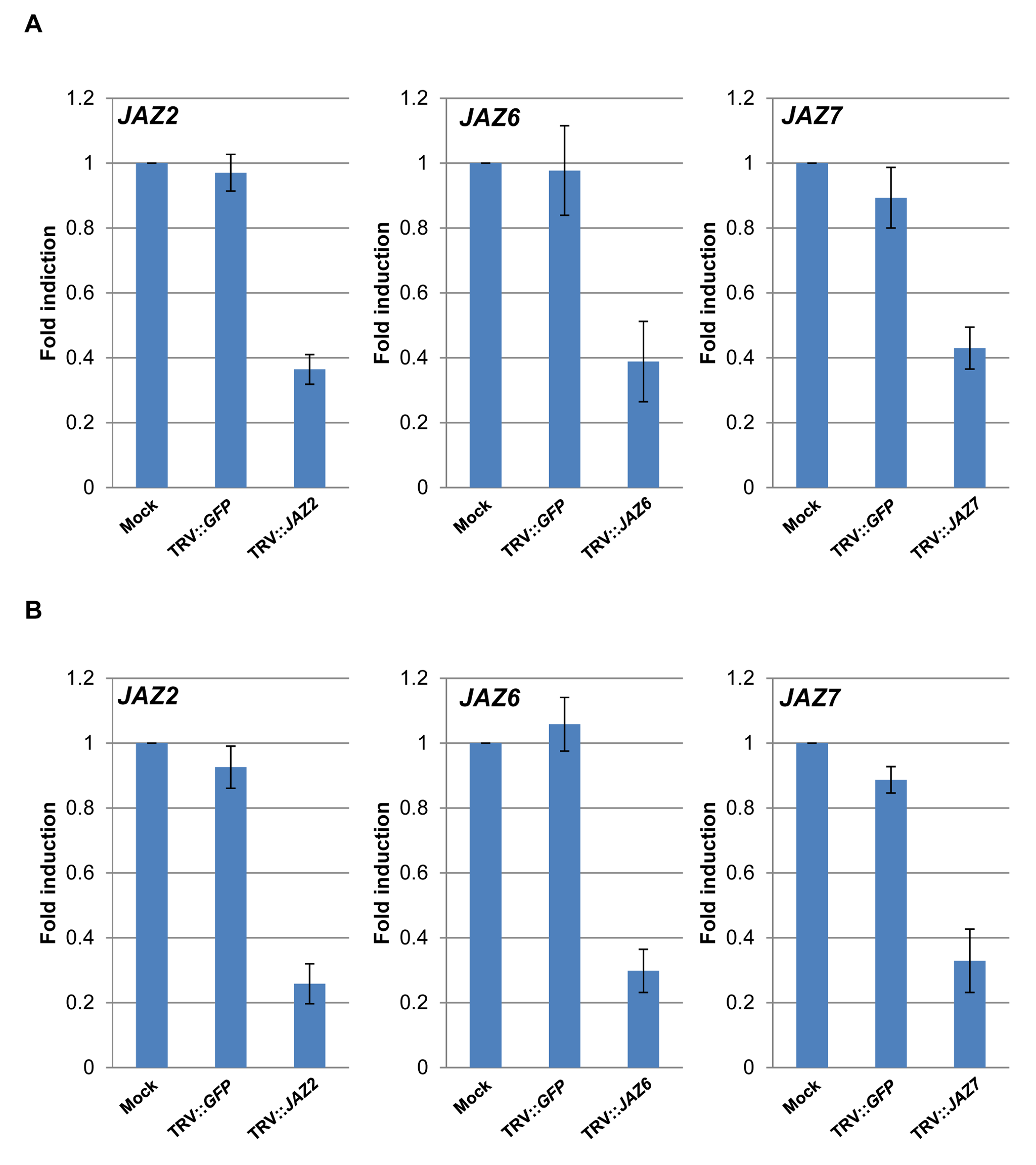

Supplement: Figure S3 — Determination of silencing efficiency of JAZ2, JAZ6 and JAZ7 in tomato and N. benthamiana . A. Relative transcript levels of JAZs in tomato. B. Relative transcript levels of JAZs in N. benthamiana. The N. benthamiana and tomato Actin were used as internal controls. (TIF) [file pone.0075728.s003.tif]

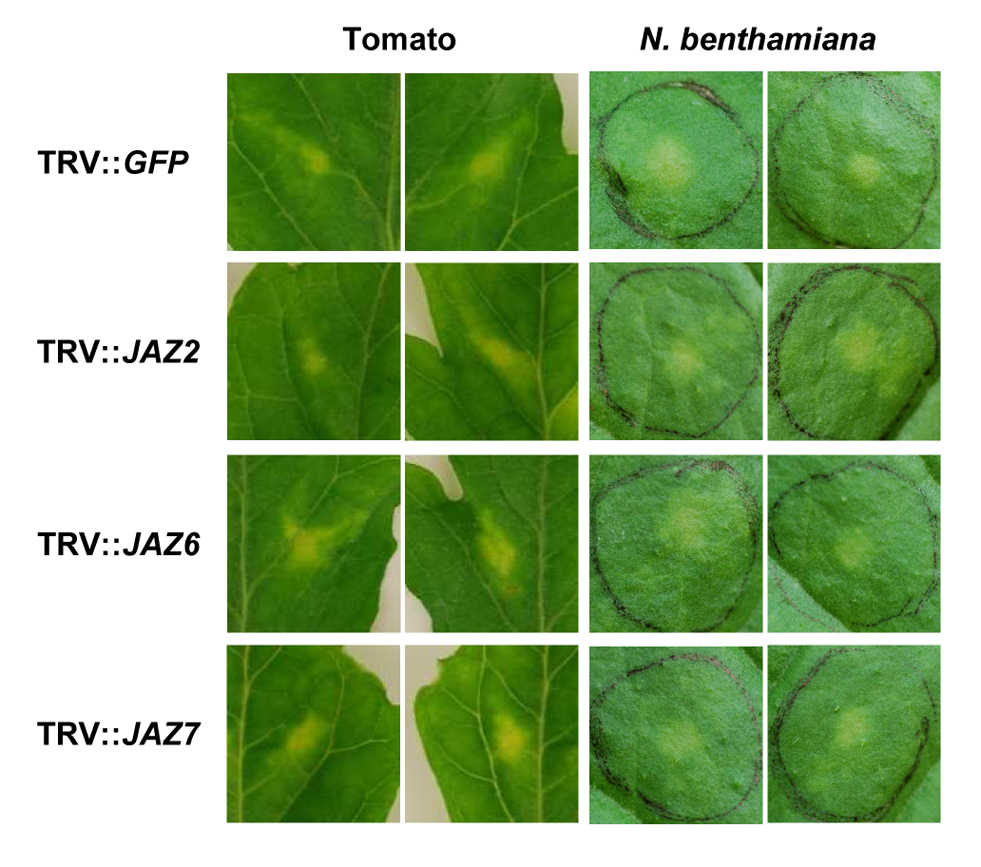

Supplement: Figure S4 — COR-induced chlorosis on control and SlJAZs -silenced tomato and N. benthamiana plants. Purified COR was applied to vector control (TRV::GFP) or SlJAZs-silenced (TRV::SlJAZ2, TRV::SlJAZ6 and TRV::SlJAZ7) tomato and N. benthamiana leaf tissues in 2 µl aliquots (2 nM), and a visible chlorotic zone was scored at 4 days after treatment. (TIF) [file pone.0075728.s004.tif]
