# Supplementary material for: Jasmonate ZIM-Domain (JAZ) Protein Regulates Host and Nonhost Pathogen-Induced Cell Death in Tomato and Nicotiana benthamiana
Source: PLoS One. 2013 Sep 27;8(9):e75728. doi: 10.1371/journal.pone.0075728 (PMC3785428; doi:10.1371/journal.pone.0075728)
Supplement: Figure S2 — Sequences of full-length ORFs for 12 tomato SlJAZ and SlNINJA. (PDF) [file pone.0075728.s002.pdf]

>SlJAZ1_Solyc07g042170.2
ATGGCTTCAT CGGAGATTGT GGATTCCGGG AGATTTGCCG GTCAGAAATC GCATTTCTCT
CATACATGTA ACTTGTTGAG TCAATACTTG AAAGAGAAGA AAGGTTCTTT GGGAGATCTC
AGCCTTGATA TGCATCGCAA TTTCGATTCA GCTGGTTCTA CTACTATGGA TTTGTTGCCG
ATGATTGAGA AATCTGGTGA GTTGGTTCAG AAATCGATGA ATCTGTTCCC TCAAGGTGGA
ATGAAGGCTG AGTCGGAACC GGAAAAGGCA CAGATGACGA TATTCTATGG AGGTCAAGTT
ATTGTGTTTA ATGATTTTCC GGCTGATAAA GCTAAGGAAA TCATGCTTAT GGCTAGTACT
AGCAAGGGAA ACAATCCTGC TAAACCATTG GAATCTGCTG CCGATTTGGT GGTTCCGAGT
TTCGGAAAAA CTTCCATCCA GGAAAATCAA ATGCCTAACC AGCCAATTGT TTCTGATTTA
CCTATTGCGA GAAGAGCTTC ATTAACAAGG TTTTTGGAGA AGAGAAAAGA TAGGCTAACT
GCAAAAGTAC CTTACCATAG AGAGGAAGCA GCAGCTCCTA AAAAGGAAGA ACACAAGGCG
CCATGGCTGG GATTGGGTGG TCAATTTGCA GTGAAAACTG AGCAATACTA G

>SlJAZ2_Solyc12g009220.1
CAGACTTGTA ACCTTTTGAG TCAATTCTTG AAGAAAAAAG GTTCTGTTGG AGATCTTAAT
AATCTTGGTA TCTACAAAAC TACTTTTGAA TCAACTGGAT CTCAACAAAC AGCTACTACC
ACCACAACTA CTATGAATTT GTTACCAATG ATTGAGAAAT CAAGTGATTC ATCGTCGTCA
TCGTCGTCCG TTGAAACAAA TCCTCAAAAA CCCATGAATC TTTTCCCTCA AGAATTTGAT
TTCTCCAAAG AACAATCCAC AAAAAAGACA GAATCTTGGA AACCTGATCA ACCAGAGAAG
GCACAAATGA CCATTTTTTA TGGTGGACAA GTCATTGTTT TTGATGATTT TCCAGCTGAT
AAAGCAAATG AAATCATGAA GTTAGCCAAC AAACAGAACC CCACAAACAA TTTCACTTAT
CCTATGATCA AGAATCAAAA AACAGCTGAT CAATCTGGTG TGAGTTTTGG TAACAAATTG
ATTCAAGAAC TTCCTAAACT GTCAATGCCA CAGCCTTCTG TTGCTGATTT ACCAATCGCG
AGACGGAATT CACTTACAAG ATTCTTGGAG AAAAGGAAAG ATAGAGTAAC ATCAATTGCA
CCATACCAAA TCTCCAACAA CAAGAAATCC AAGAATGAGG ATAACAAAGC ATGGTTGGGA
TTAGGTGCTC AATTTGTTAA AACTGAGCAA TATTTCTAG

>SlJAZ3_Solyc03g122190.2
ATGTCGAATT TATGTGACGC TCGCCGGAGG AATGGAAACG GCAAGGCGCC GGAGAGATCA
AGTTTCGTAC AGACTTGTAA CTTGTTGAGT CAGTTTATTA AAGGGAAAGC TACTATTAGA
GATCTGAATC TCGGAATCGC CGGTCAGCCG GAAGCTGCAG GGAAAACTGA AACTGCAACT
ATGGATTTAT TAACAGTCAT GGAAAAACCA TCTATTGATC TAACAAAAGA AGAACACAAA
TCCGTAGATC TTGTTACAAC AGAGTCTTCT AGAGAGAAGG AGGCCGCTGT AAATGAGCCC
AGCACAAGCA AAGAGGCACC AAAGGAGCCT AAGGCAGCCC AATTGACTAT GTTCTATGAT
GGTAAAGTGA TTGTTTTTGA TGATTTCCCT GCTGACAAAG CTAGAGCAGT GATGTTATTG
GCTAGTAAAG GATGTCCTCA GAGTTCATTT GGGACTTTCC AGGCTATAAA CATCGACAAA
ATTAACACAT GTTCTCCTGC CCCTGCTTCT TTGACAAGTA ATAGAACTGA TTCTGTTGCA
CCACAGCAAC AACACCTCCA GATTAAGCCA GACTCTTGCT CTGCTGCACC GCAACAGCAC
AAGCACAATT CGCCCCCACT CCATGTTTGT AGTAGTACTA AAACTGATCA GCTTAAGCTC
GGATCAGTTT CATCTGCACC CTTAGTAGAG CAGGAGCAAC ACAAGCAAAT CCAGTCACAG
GCTGCCGAAA TTAGCAGCAG CTCCGAGCTA CCTATCGCAA GAAGATCTTC ACTTCATAGG
TTTCTCGAGA AGAGGAAAGA TAGGGCAACT GTTAGAGCTC CATACCAAGT TGTACGTAAC
AATCCGTTGT TACCATCTTC TTCGAATACT AATGGTGAAT CATCTTCTAA GGATAGCGAA
GATCAGCTCG ATCTCAATTT CAAGTTATAG

>SlJAZ4_Solyc12g049400.1
ATGTCAAATA GGCAACTTTG TTCATTAGAT AGTGAGAAAT CACATTTGAT GAATACTTGT
AATTTGTTGA CTCAATTTTT CAATGGAAAA GCAAATATCA ATGATCTAAA TCTTACAATT
AGTAACAATG GAGAAGCCAA AGCCTCAGCA ACAAAGGATT TGTTAACCAA CATGGAAGAA
TTATCAACTA AGACAACAGA ACAAGACCAG AAATTAATAG ATCATGTTCC TAAAAGTGCA
ATAAATAAAG CCAGCGGCAG CAAAGAGATA CCACATAAGG AGCAAAAGCT AGCCCAATTA
TCTATATTCT ATGGGGGTAA AGTGGTGGTT TTTGATGATT TTCCAGCAGA GAAAGCCAGA
GCAGTGATGT TATTAGCTAG CAAAGGAATC TCTAACAACT CTTGTGCCAT TTTTCAAACC
CCCACCACCA CTCAGACTAA TGGATCCAAC AACTTTGATT TACCTATTGC AAGGAGATCT
TCACTTTATA GGTTTCTTGA GAAGAGAAAA GACAGGGACA CGGCTAGAGC GCCATACCAA
ATGCATAATC CATTGCAATC ATCTTCAAGG ACTCGTGGAG ATCATTTTGA TCTCAATTTC
TAG

>SlJAZ5_Solyc03g118540.2
ATGGAGAGAG ATTTCATGGG GTTGACTGTG AAGCAAGAAG TTCTTGAAGA ACCTATAGAT
CCAGCACCTT TAAGAAGTTC AGCAATGCAG TGGTCATTCA CGAACAACGT CACTGCTCAT
CCTCAATACC TTTCTTTCAA GAGTGCTCCA GAGGATAAAC CGAAAATTGG TTTTGATTCT
CTTGCATCAA CTGGATTGGT GACTATAACC ACAACTGAAG CTGTTGACTC GAGTCATCGG
ACATACTCTG ATGTCACACA GAATCTGATA ACTACTGTAA ATCAACTCCC TGGTGCTGGA
GCTCTGGTTG TTAGCCCCAT TTCAGCTGTT CCGTCTAGCA GCATTGTCGT GGGCACCACT
GATTTAAGGG GTGCTCCCAA AACTCCCCCA GGTCCTGCCC AGTTGACCAT CTTTTATGGT
GGTTCTGTCT GTGTGTATGA CAATGTTTCA CCGGAAAAGG CTCAAGCTAT TATGTTACTT
GCTGGAAATG CACCACCTGT TACACCAAAT GCAACATCTA CTCTATCTCC AGTTCAGGCA
CCTATACCTA AGTCCTCGGC CATAGACTCT TTTGTTGTAA ATCAGTGCCG TAATACAACA
CCTACACTTG CCAGCCCCAT TTCTATAACA TCCCATGGTG GCGCGCAAGC TGCCAGAGTG
TCTACTACTA CAAATGGAGT AACTATTATC AAATCAATAG GAGTTCTTCC ATCTCCTTCT
CTTAAAGCCG AGCCTTCCAA AGTTACCAGT TCTGTAGGAT CTTTTCCTGC CTCCCTCGTT
CCATCAGCTG TGCCTCAGGC GCGCAAGGCA TCATTGGCTC GATTCTTGGA GAAGCGCAAG
GAAAGGGTAA TAAGTGCATC ACCTTATCCT CTCAACAGCA AGCAATCCCC AGAATGTAGC
ACTCCTGAAC TTGGAAGTAG AAGCCTCTCT ATGAATTCTT CAGGCTCTTG TCCTCCCCAC
ATAATCAGTT TGGTCAAGTA G

>SlJAZ6_Solyc01g005440.2
ATGGAGAGGG ACTTTATGGG ATTGAATATC AAAGATTCTT TACTTGTAGT CAAGGATGAA
CCTGTTGAAA GCTCAAAAGA CTCTGGGTTT CGCTGGCCAA TGTCGAGCAA GGTTGGTGTA
CCTCATTTCA TGTCCTTGAA CTCTGCTCAA GATGAGAACA CCTTCAAAGC TCTATCTGCC
ACAGATGGAG TCGATGCTGG TCTCAAACGT CAGCCTGGTG AACTCCAGAT GAAGCAAGTT
CTTGGTGGAA TTCCTGTTAC AGCTCCTCAT TCAATGCTTC CATCGCGTGG CTCTGTGGCT
GGAACAACCG AACCTTGGTT TAATTCCAAG GGTTCTGCAG CACCTGCTCA ACTGACCATC
TTCTATGGTG GGATGGTCAA TGTCTTCGAG GATATCTCCC CTGAGAAGGC ACAGGCTATT
ATGTTTTTGG CTGGACATGG CTGTGCTCCA CCTAATGTGG TGCAGCCAAG GTTTCAACTT
CAGGCATCTG CATCGAAACC TGCTGCTGCA GATGGTGTTT GTGTGAACCA AACCCCAAAC
ATGCTGCCTG CCTCGGGTCT TTCTAGCCCT ATGTCCGTTT CTTCCCATCC CATTGGTCAA
TCTGATGGCA GTTCTGGAAA CAAAGATGAC ATGAAGATGT CTAAAACTGC AAACATTTCA
GTGACTCCCC ATGTCAAATT GGACACTTCA AAGATTGTGA CATCACTAGG ACCTGTTGGG
GCGACTACCA TAATGACAGC AGGTATGGCG TCGGTTCCAC AAGCTAGGAA AGCATCTTTG
GCTCGTTTTC TGGAGAAGCG CAAGGAAAGG GTGATGAACT TAGCACCATA TGGCCTCAGC
AAGAAATCGC CTGAGTGCTC CACCCCCGAG TCTAATGGAG TTGGTTTCTC TGCAACTTCC
ACTCCTCTGT TAGCCGGTAA GGAGACCTAG

>SlJAZ7_Solyc11g011030.1
ATGGATTCAA GAATGGAGAT AGATTTTATG GACCTCAACA GCAAACCAAA ATTATCAGAA
ATGGAGAAGC AACACAAAAA AGTATCTGGA ATGAAGTGGC CATTTTCATT GGCTGATTTA
GCTACTCACC ATGAACACAC ATTTTTTCAG AATTACAAAT CCACCCCAAT AGTTTCCATT
AATTCAAAAA ATTCATCCCT AAACAATTAC AAATCCACCA TTGACCCCCA ATACTTTAGA
GGGACTTTTC CTCTATTAGC AAAAACAAGC ACTTATGACT CAAGGAAAAA TTATGACAAT
TTGAGTCCAA ATGAGTCAAC ATTGACCATA TTCTACATGG GTGAGGTCCA TATTTTTCCG
GGTATATCAC CAGAAAAGGC TGAGCTTATA ATTGACCTGG TTTCTAAATC AACAACTCTC
CACATGGATG AGATTTTAGA AAAAGTGATG AATAAAGAAA AATATGAAGA AAATAAATCA
GACCCTTCAA ATGCATCCAC AAATTATGCT AAAGGAGCAC TTGCTATGGC TCGTAGAGCA
ACTCTTGCAC GATTTTTGGA GAAGAGAAAA CATAGATTGA TCAAAGCTAG GCCGTATCTA
TATGGGGAAA ATTTATCAAA GTTTCCCTTT GATATTCAAC AACAAGAAGA AGAAACGGCG
TCGTCAAGCG TTCATTGGGA AAACTAA

>SlJAZ8_Solyc06g068930.1
ATGCATTGGT CATATTCTAA CAAGGCTCAT CCTCAATACC ACCTTTCTTT GAAGGACCAA
GAGAATATTA TTATTAATAA TAATAATAAT AATAAGCCTA AGATTGGTTT CGAATCTCTT
GCATCAGCTG GATTAGTGAC GATAACCACA ACTACTGAAC TATTTGACAC AATTCATAGA
CCATATACTA CTCAATTTGG TGCACATCAT GTCCCAACAC GCAATGGTGT TGTGGGCACC

ACTGAATTGA GGGGTACACC TAGACCATCA CCAGGACCTG CTCAATTGAC CATGTTTTAC
GCTGGTTCTG TCTGTGTTTA CGATAATATT TCACCAGAGA AGGCTCAAGC TATTATGTTA
CTTGCTGGAA ATACACCAAT TAGTACAACA ATTCGAAATT CTCCTTCTCT TGATCATCAT
CATCATCATC ATCATAATAA TAATAATAAT AATAATAATA ATAGTACAAA TGAAACAACA
ATTATCAGAT CAATCGGAGT CCTAAAATCC CATGAGCTAT CGAAAATTGT TACTTCTCAA
GAATCTCGTC AACCTCCCAA TCATAACTTA TCAGCTGTTC CTCAGGCTCG CAAGGCATCC
TTGGCTAGGT TCTTGGAGAG ACGCAAGGAA AGGGTAGTGA GTGCATCACC ATATGGAAAT
GGCAAGCAAA GCTCACAACA TATGATGAAC TTTACAATAA ATTCCTCAGG CTCAAGTACT
TCACTTCCTG CTGCAAATTA G

>SlJAZ9_Solyc08g036640.2
ATGAGAAGAA ATTGTAATTT GGAGCTCACT CTTATGCCTC CTTCTATTTC AGATAACTTT
TCTTCTAAGA ATTGCACTAC GGAGGATCAA CAATTGGAGA ATAAGCAATC GCAACAGCTA
ACCATATTTT ACCATGGAAA ATTTGTGGTT TCCGATGCTA CTGAGCTTCA GGCTAAAGCT
ATAATATATC TTGCAAGTAG AGGAATGGAG ATGAAAACAA ACAAGATGTC TGAGCCTTCA
TCACCATTAT TACAACCTCA AACTGTGAAG AAATCTCTAC AAGGATTTCT ACAAAAACGA
AAAAAAAGAG TTCAAGCAAC TTCGCCATAT CACAAATAG

>SlJAZ10_Solyc08g036620.2
ATGAGAAGAA AGTGTAATTT GGAACTCACT CTTTCTCCTA GCAACTTGTT GATGGAGGAT
AAAAGATTAG AGAATGAGCA ATCACAACAG CTAACTATAT TTTACAATGG AAAATTTGTT
GCTTCTCATG TTACTCAGCT TCAGGCTAAA GCTATAATTT ATCTTGCGAG TAGAGAAATG
GAGGAGAAAA CAAATAAGCT GTCTGAGCCT TCATCACCAT TATTACAACC TCAAACTGTG
AAGAAATCTT TACAGAGATT TCTACAAAAA AGAAAAAATA GAATTCAAAT AACTTCTCCA
TATCATCACT AG

>SlJAZ11_Solyc08g036660.2
ATGAGAAGAA ATTGTAATTT GGAGTTTAGG CTTATGCCAC CTTCTCTTTC TACTTTTTCT
CCTAACATTT GCAGTAATAA TAATACATCC TCCTATTTTT CAATGGAGGA GGATAAAGAA
AGCACAGAAT TAGAGCAGAA ATCTGAGCCG TTAACCATAT TTTACAATGG AAAACTTGTG
GTTTCTCATG TTACTGACCT TCAGGCTAAA GCTATAATAT ATCTTGCAAG TAGAGAAACA
GAGGAGAAAA CAAACAAGAG TCTGTCACCA ATATCTGAGC CATCATCACC ATTATTACAA
CCTCAAACTG TGAAGAAATC TCTACAAAGA TTTCTACAAA AAAGAAAAAG TAGAACTCAA
ACAACTTCGC CATATCATCA CTAG

>SlJAZ12_Solyc01g009740.1
ATGTCTTCAG GTACTGATAA TGTGATTGGA AATACCCATC CCTATGAAAT TCTGAAGAAT
GAAACAACAA ATATTGTGAC TCTGAGTTTT ATCTGCGATG AAATAGTTGA GATTTTTAAT
GTCTCAAGGG ATAAGGCTGA GGAAATTCTA AAGTTTGCTG ATATGTCAAA AGTTGTGAAT
GACTGTTCAT CAAAAAAACC ATTGGGTTTG GAAACACAAA GTCAAGGTAA AGGTAAGGAG
AAACTCTCAA CAGCGAGGAG AAACTCTCAA CTTAGATTCT TGGAGAAGCG AAAAGAGAGG
ATTTGTGATG TCTTGGAAAA TGGCATGAAA ATCGTGCTTA TGACTATTGC GTGCAATGGA
AAGGTTGCAA ATTTCAATGT CTCCAGTGAA AAGGCGAAGG ATATCCTAAT ATTTGCAGAA
TTGTTAAAAG TTACGGATGT CATTCCACCA ACAAAAAGCC AATCAAATGT GGAAACATTC
AGTGGAGGTA GATTTTGGGA GAAGCAATAT CCGAGGCTTA ACTCTTGCTT GAAAAATGGC
ATAATAATTG CGCCTTTGAC TATTTGCTAT GATGGAACGA GTGTGAGTTT CAATATCTTC
AGTGATAAGG TGGCAAATAT TCTAGAGTTT GCAGAAAAGT TAAAATTTAG AGATGCAGTT
TCATCTGAAA AACAATTATT CGTGGAAACA TTAAGTGGAG CTCAACCTTT GCCTAGGAGA
AACTCTATGC TTGGATTCTC GGAGAAGGGA AAAGAGAGGG TTTGTGGTGG CTCGAAAAAT
GCCAAGGAAA TTTCACTTTT GACTATTTGC TACAGTGAAA TGGTTGCATC TTTTTATGTC
ACTAGTGACA AGGCGCATGA TATTCTAAAA TTTGCTGAAA CATCAAGAGT TAGGAATGAC
TTTTCTCCAA ACTTTGGAGA TTTATCATTG ACAAGGAGCA TCTCCTTGCC TAGATTCTTG
GAGAAGCCAA AAACAAGGCT TGACTACATT ATGCCTATGA CTTTTTTCTA CGAAGGAAAC
ACCACTACTC TCAATGTCTC TATTGATAAG GCAGTAAATA TTCTAGCGTT CGTAGAAAGG
TCAAATTTTG GTGATGCATG TCCATCTGGA AAAAAATTCC CCGCGGAAAT ATTAAGTGGA
GATCAGCATT TGGCTAGGAG AAACTCTGTG CTTGGATTCT CAGAGAAGCA AAAAGATAGG
CTTTGTGATG GCTCGAAAAA TGGCTTGAGA ACTGCACTTT TAACTATTTG CTACTGGGGA

ATGGTTGGAA ATTTCAATGT CTCCAATGAT GAGGCGAATG AAATTCTAAA GTTTGCAAAA
AGGTCAATAA CTAATGATGT ATTTTCATCA AATAGTGGAG ATCTATCATT GCCGAAGATA
AACTCTTCGC TTAGATTCTC CGAGAAGCCA CAAATAAGGC TTGGTTGTGA GTCGGAAAAT
GGCGTTAAAG TTGAACCTTT GACTATTTTT TATGATGGGA AGATTGTGGT TTATGATGTA
TCCATTGAGA AGGCAACAAA TATTCTTAAG TTTGTAGAAA GAGATGCAAT TTCATCTAAA
AAGGAATTTT CCATGGAATC ATTAAGCGGA GATCTTCATT TGGCTAAGAG AAACTTTATG
CATGGAGTTT TGGAGAATCG AAATGAGAGG TTTTGTCCTC CGGGATTCGA AGACATAAAA
ATTGCGCCTC TGAGTATCAT CTACAATGGA AAACTTGCGA TTTTCGATGT CCGGAGTTAC
AAGGTGGATG AAATTCTAAA GTTCGCAGAA TCGTCAAAAC AACAAACCTT AAGCCAAGAT
GTATTGACAA CGAGAAAATC CTCAGTTAGA TTCTTGGAGA AGCGACAAGA GAGGATGACT
ATGGTGTCAC CTTATGGTTT TCCCCATGCT GCCCCTGAAA ATAAGAAATA G

>SlNINJA_Solyc05g018320.2
ATGGACGAAA ATGATCTTGA TCTAAGCTTG GGCCTGCCCT GTGGTGGGGT GGTTGCGTCA
GAGAAAAGTA AAAGTGGGAG CTCATCGGAT TCCAAGGTTG AGGAAGTTGA TAGAGATGGA
AAAGTGATTA ATGATTTCAA GAACTTTCTG GATGGAGGCA CTAGCAGCCA AAAGCATGAT
TGTGGTGTCG GTTCTCAGAG AAGTGATTCA ACAAAACATG GTGGGAACTT GCTTTCCAGC
ACTAGTGCTG ATGCAGATGC TTCTAAAAAG TTAAATAGTG GAGGATTCTG GGTTCCAAAT
GATAATAGAC CTATAGAAGT TGAAGAAGAA AGGAGAACTG AAGTGGGTGA AAAGCGTAAA
AATTTGTTCC GGGAGTCAAG TCAACAAAAG AAGCATGAGA GAGAAGCTCA TCATGTCGAT
ATGCATGACA AGACAAGGGC ATCACACATT TCAATAACAA CAGATGATGG TTCGACTGCA
GAAAATGATG ATGTAGCTGA TTCTGAAACT GTGGGTTCAA CTTCCAGGCA AATTTTGCAG
CATGATGAGA ACTCTAAAAG ATTTGTTGGA AGTAGTGCTG AGGTTCATAA GGAGCTTCGT
GGTGTTTCTG ATTCAAGTGG TGTAGAATTA CTTGGACAGA GAAGGTTTAC CATTTCTTCT
GAAAAGGATG TTAAGTTCGG TAATACGCCA TACAGTACCC CATTCCAAGG CCAATCAATA
AACATCATGA ACCTACCATA CTCTATGCCT CTGAGAGATT CTAACCCTGG TAATACAGCA
AGTACGACTG GTTATGCAGT TCCTGGCATG ATGCAAGTAA TAGCTACCAC TAGTGGAGAT
AGACCTGGAG CCCAGCCTGT CATACCTACT AATTTGCCAT TGATGTTTGG CTACTCTTCC
ATACAGCTGC CAACATTGGA GAAGGATAAT TCCCGCGGTG CAGCTTCTCA TCTTCAGCAG
CTTCACCCTT CCTATGGACG AGGTTCCTTG GGCTCAGACA AGCATAAAGA TGGACCAAAT
ATTTCTCAAG CTACGTTGCC GATTATTGCA CACAAGTCTT CTGAATCTGT ACAATATGAT
GGGAGGGCAG TGGAGCATGT GAAAGGCAAT GGGAGACAGC ATAACGCGGA AGAAACTTCC
ACTTCTCGAG TGGAAGAAAA TGTTAAAGGT AGCAACATAA GCTTCAGGTC AAAAGACCCT
CCTGAGCAGC CGAGAGCTGA AGCAGTTCCT TCAGAATTTG CAACTATAAG GCCAGGTCTT
GCTGCAGATC TGAAATTTGG AGGATCTGGT TCCTATCCGA ATCTACCATG GGTTTCAACA
ACTGGTCCAG GTCCAAATGG TAGAACAATA TCTGGTGTTA CTTATAGATA CAATCCCACC
CAAATCAGGA TTGTTTGTGC TTGTCATGGG TCTCACATGT CACCGGAAGA GTTTGTGCGG
CATGCTAGCG AAGAGCAAAC TAGTCAAGAA GGTGGTGCTG GTGTTTCATC GTTTCCAAGT
AGCAATCCTG CTGCCTCTGC ACAAAGCTGA
